# Oleic Acid Improves Goat Sperm Quality by Enhancing the MBOAT2/ACSL3 Pathway to Attenuate Ferroptosis

**DOI:** 10.3390/ani15223258

**Published:** 2025-11-10

**Authors:** Wen Bi, Zhendong Zhu, Adedeji O. Adetunji, Shengyan Zhao, Dongping Ma, Xin Kou, Lingjiang Min

**Affiliations:** 1 College of Animal Science and Technology, Qingdao Agricultural University, Qingdao 266109, China; 19011590820@163.com (W.B.); zzd2020@qau.edu.cn (Z.Z.); 2 Department of Agriculture, University of Arkansas at Pine Bluff, Pine Bluff, AR 71601, USA; adetunjia@uapb.edu; 3 Shouguang City Animal Husbandry Development Center, Weifang 262700, China; sdsgzsy@163.com (S.Z.); sgylmdp@163.com (D.M.); 4 Hongde Livestock Farm, Yingli Town, Weifang 262700, China; kouxin2004@126.com

**Keywords:** ferroptosis, oleic acid, oxidative stress, sperm

## Abstract

Exposure to low temperatures during goat sperm preservation induces oxidative damage and triggers ferroptosis, a regulated form of cell death, thereby impairing sperm quality and fertility. This study aims to elucidate the protective mechanism of oleic acid (OA) on goat sperm during cryopreservation through the regulation of ferroptosis pathways. Different concentrations of OA (0.125 mM, 0.25 mM, 5 mM, 1 mM, and 2 mM) were added to a yolk-containing extender. Following chilled storage at 4 °C, sperm motility parameters, membrane and acrosome integrity, mitochondrial membrane potential, and other physiological parameters were systematically evaluated. Oxidative damage was analyzed by quantifying ferrous ion (Fe^2+^), malondialdehyde (MDA), lipid peroxidation (LPO), and reactive oxygen species (ROS). Western blotting was employed to assess the expression of key regulators of the ferroptosis pathway, including ACSL3, ACSL4, GPX4 and SLC7A11. The results showed that sperm motility in the 1 mM OA treatment group was significantly higher than that in the control group (*p* < 0.05), with improved membrane and acrosome integrity, as well as significant mitigation of mitochondrial damage. Moreover, OA effectively suppressed ferroptosis by reducing Fe^2+^ accumulation and inhibiting MDA and LPO production. Mechanistically, OA may regulate membrane phospholipid composition by competitively incorporating monounsaturated phosphatidyl ethanolamine, thereby reducing ACSL4-mediated lipid peroxidation. These results suggest that oleic acid improves sperm motility by inhibiting ferroptosis during liquid storage at 4 °C.

## Simple Summary

Exposure to low temperatures during goat sperm preservation induces oxidative damage and triggers ferroptosis, a regulated form of cell death, thereby impairing sperm quality and fertility. This study investigated whether oleic acid (OA), a naturally occurring monounsaturated fatty acid, can mitigate these effects during chilled storage. Supplementation of semen extenders with varying concentrations of OA revealed that 1 mM OA significantly enhanced sperm motility, membrane and acrosome integrity, and mitochondrial activity after preservation at 4 °C. Also, OA reduced oxidative stress markers—including reactive oxygen species (ROS), malondialdehyde (MDA), and lipid peroxidation—and modulated the expression of ferroptosis-associated proteins (ACSL3, ACSL4, GPX4, and SLC7A11). Moreover, OA suppressed iron accumulation and alleviated ferroptosis in an RSL3-induced damage model. These findings suggest that OA exerts potent cytoprotective effects by modulating lipid metabolism and inhibiting ferroptosis, rather than functioning solely as an antioxidant. This study provides new insights into improving sperm chilled protocols and may have broader implications for assisted reproductive technologies in livestock breeding and genetic resource conservation.

## 1. Introduction

Goat artificial insemination has become an indispensable tool for improving reproductive outcomes by enabling more precise control over genetic transmission [1,2]. In livestock breeding, chilled storage in particular—in combination with protective additives—plays a crucial role in extending sperm lifespan [3,4,5]. However, sperm quality deterioration caused by the chilled storage process remains a critical factor limiting fertilization efficiency. Among these technologies, sperm chilled storage is a core method for the long-term preservation and intergenerational use of high-quality genetic resources. Unlike bovine and porcine sperm, goat sperm is particularly susceptible to oxidative imbalance during chilled storage, a vulnerability attributed to the unusually high proportion of polyunsaturated fatty acids (PUFAs) in its plasma membrane [5]. Structurally, low-temperature-induced oxidative stress destabilizes membrane architecture through phospholipid peroxidation, while functionally impairing sperm dynamics by disrupting flagellar beating patterns [6,7,8]. These subcellular injuries reduce post-thaw sperm survivability and compromise their ability to penetrate oocytes during in vitro fertilization procedures [9].

Exposure to thermal fluctuations under chilled gradients rapidly accelerates the accumulation of reactive oxygen species (ROS) in sperm cells [10]. As primary targets of ROS, PUFAs undergo peroxidation, generating toxic compounds such as 4-hydroxy-2-hexenal and malonaldehyde [11,12,13]. These products covalently bind to membrane phospholipids, thereby disrupting membrane homeostasis and selective permeability, ultimately resulting in reduced sperm motility and fertilization potential [14]. Ferroptosis is a distinct regulated cell death process, marked by uncontrolled PUFAs peroxidation within cellular membranes [15,16,17,18]. The likelihood of ferroptosis is closely influenced by the lipid composition of membranes: enrichment of polyunsaturated fatty acids, relative to monounsaturated species, predisposes cells to oxidative chain reactions [19,20]. This mechanistic insight forms the basis of a novel therapeutic paradigm: selective enrichment of MUFAs may interrupt peroxidation propagation, thereby attenuating ferroptosis signaling and preserving membrane integrity [21]. When the endogenous antioxidant defense system, such as glutathione peroxidase 4 (GPX4), fails to eliminate excessive ROS, an iron-dependent lipid peroxidation cascade is triggered leading to ferroptosis, a form of cell death characterized by mitochondrial cristae collapse and lipid peroxide accumulation [22,23]. Emerging studies have revealed that ferroptosis is the predominant form of regulated cell death during chilled storage of goat spermatozoa. Pharmacological inhibition of ferroptosis using specific inhibitors has demonstrated substantial cytoprotective effects, effectively mitigating oxidative damage by lipid peroxidation [24,25].

Recent studies have shown that the exogenous monounsaturated fatty acid oleic acid (OA) can inhibit ferroptosis by regulating membrane lipid metabolism [26]. ACSL3 primarily facilitates the activation of monounsaturated fatty acids (MUFAs), including oleic acid, and is involved in the biosynthesis of both membrane phospholipids and neutral lipids [27]. Through these processes, it supports lipid droplet assembly and helps preserve cellular membrane integrity. Conversely, ACSL4 shows a substrate preference for polyunsaturated fatty acids (PUFAs), driving the production of PUFA-enriched phospholipids that act as precursors for lipid peroxidation. Mechanistically, OA can be incorporated into phosphatidylethanolamine within membranes, displacing polyunsaturated fatty acids and consequently suppressing the ACSL4-dependent lipid peroxidation cascade [27]. This mechanism has been well-documented in both cell culture models and nematodes [28]. Furthermore, mouse studies have shown that OA significantly ameliorates ferroptosis-induced lipid peroxidation in hepatic tissue [26]. This study aimed to elucidate the molecular mechanism by which OA activates ACSL3 to regulate sperm ferroptosis and exerts chilled storage effects during low-temperature preservation, thereby providing a novel strategy for optimizing chilled storage protocols.

## 2. Materials and Methods

### 2.1. Materials

Routine chemicals and reagents were obtained from Sigma-Aldrich (Shanghai, China), Vazyme Biotech (Nanjing, China), and Jiancheng Bioengineering Institute (Nanjing, China).

### 2.2. Semen Collection

This study involved 10 goats, each approximately two years old. Semen was collected once weekly. In each replicate, ejaculates from the 10 goats were collected, pooled, and evenly allocated to six treatment groups. The experiment was conducted in three independent replicates; for each replicate, the semen from these 10 goats was pooled prior to use to minimize individual variation.

### 2.3. Semen Processing

Sperm motility was evaluated using a computer-assisted sperm analysis (CASA, HT, Beverly, MA, USA) system, and only sperm samples demonstrating a motility rate above 80% were included for subsequent processing. To reduce individual variability, the ejaculates were pooled and thoroughly mixed. The pooled semen was then divided into six aliquots, each diluted with a yolk extender containing OA 1:9 (*v*:*v*) at 0, 0.125, 0.25, 0.5, 1, and 2 mM, the sperm concentration was 1 × 10^8^ sperm/mL at this stage, and placed in a constant temperature incubator at 25 °C for 30 min, and then placed in a constant temperature incubator at 17 °C for 30 min. The diluted semen was then placed in a 10 °C constant temperature incubator for 40 min, followed by a 4 °C constant temperature incubator. All treatment groups were set up in triplicate and sperm motility parameters, acrosomal and plasma membrane integrity, and mitochondrial activity were assessed at 24, 48, 72, and 96 h of storage. Concurrently, samples were collected and processed at each time point for subsequent analysis using commercial assay kits and Western blotting.

### 2.4. Experiment Design

Experiment 1 aimed to evaluate the protective effects of oleic acid (OA) during chilled storage of goat spermatozoa and to determine its optimal concentration. OA was added at 0.125, 0.25, 0.50, 1, and 2 mM [29]. Sperm motility, acrosomal integrity, and plasma membrane integrity were assessed at 24, 48, 72, and 96 h to identify the optimal dose. Oxidative and ferroptosis-related parameters, including ferrous iron (Fe^2+^)(E-BC-K881-M, Elabscience, Wuhan, China), malondialdehyde (MDA) (EATP100, Nanjing Jiancheng Institute of Biotechnology, Nanjing, China), lipid peroxides (LPO) (A160-1, Nanjing Jiancheng Institute of Biotechnology, Nanjing, China), and reactive oxygen species (ROS) (M36008, Thermo Fisher Scientific, Shanghai, China), were measured, and the expression of ACSL3 (DF9606, AffinitY, Beijing, China), ACSL4 (ABclonal, Wuhan, China), GPX4 (ET1706-45, HUABIO, Hangzhou, China), and SLC7A11 (A2413, ABclonal, Wuhan, China) was analyzed by Western blotting at 72 h.

Experiment 2 aimed to verify the role of OA in RSL3-induced ferroptosis during chilled storage. Based on preliminary tests, 10 nM RSL3 was selected as a ferroptosis inducer, and sperm were co-incubated with 1 mM OA and 10 nM RSL3 at 4 °C for 72 h. After incubation, sperm motility (CASA) and the levels of Fe^2+^, LPO, and MDA were determined.

### 2.5. Measured Parameters

A comprehensive assessment of sperm function and biochemistry was performed, including: computer-assisted sperm analysis (CASA) of kinematic parameters (velocity (VCL, μm/s), straight-line velocity (VSL, μm/s), average path velocity (VAP, μm/s), linearity (LIN, %), straightness (STR, %), wobble (WOB, %), beat cell frequency (BCF, Hz), progressive motility (%), and total motility (%)); evaluation of acrosomal integrity, sperm viability, and mitochondrial membrane potential; measurement of intracellular reactive oxygen species (ROS), malondialdehyde (MDA), and lipid hydroperoxide (LPO) levels to determine oxidative stress stat us; analysis of MBOAT2 enzymatic activity along with ferrous and total iron content; and finally, immunofluorescence localization of MBOAT2 and ACSL3 combined with Western blot analysis of ACSL3, ACSL4, GPX4, and SLC7A11 protein expression.

### 2.6. Preparation of the Stock of BSA Dissolved Oleic Acid

BSA (bovine serum albumin) (ST025, Beyotine, Shanghai, China) is a protein derived from bovine plasma. A total of 0.6 g BSA was dissolved in 3 mL PBS and thoroughly mixed to obtain a clear brownish solution. Then, 0.04 g NaOH was dissolved in 10 mL of deionized water. 3 mL of the NaOH solution was added to 19.04 μL of oleic acid, and saponified in a 75 °C-water bath till it turned colorless and clear. Finally, the saponified OA solution was mixed with BSA and incubated at 55 °C for 30 min to obtain 10 mM OA. To control any potential effects of BSA, we included it in the control group at the same concentration as in the oleic acid-treated group.

### 2.7. Evaluation of Sperm Motility by Computer-Assisted Sperm Analysis (CASA) System

Sperm motility traits were assessed using a CASA system. After pre-warming, 4 μL of sperm suspension was placed on a glass slide, and five random fields were selected under a 40× objective lens for observation and analysis [30]. Sperm motility parameters were recorded and analyzed. Imaging parameters were set at brightness 60, contrast 750, and light intensity 1000, with threshold settings of VCL > 80 µm/s, VSL > 50 µm/s, and VAP > 25 µm/s. Progressive motility was defined as spermatozoa with STR > 80%, and total motility was calculated as the sum of progressive and non-progressive motility [31]. Recorded parameters were curvilinear velocity (VCL, μm/s), straight-line velocity (VSL, μm/s), average path velocity (VAP, μm/s), linearity (LIN, %), straightness (STR, %), wobble (WOB, %), beat cell frequency (BCF, Hz), progressive motility (%), and total motility (%).

### 2.8. Evaluation of Acrosomal Integrity and Viable Spermatozoa Preserved

According to previous studies, sperm plasma membrane integrity and acrosome integrity were assessed using a live/dead sperm motility assay kit (L-7011, Thermo Fisher, Shanghai, China) and fluorescein isothiocyanate-peanut lectin (L-7381, Sigma-Aldrich, Shanghai, China), respectively (include reference to the previous studies you referred to) [32]. Methanol-fixed sperm samples were incubated with fluorescein isothiocyanate-labeled peanut agglutinin (FITC-PNA) solution at 37 °C for 20 min to evaluate acrosomal membrane integrity. Spermatozoa exhibiting negative FITC-PNA staining were identified as cells with intact acrosomes, while those that were PI-negative were considered viable spermatozoa with intact plasma membranes [33]. Subsequently, flow cytometry (BeamCyte, Changzhou, China) analysis was performed, with 20,000 events recorded for each sample. And using FlowJo (v10.8.1) to analyze raw data. Each treatment group was performed with three independent biological replicates (*n* = 3).

### 2.9. Evaluation of Mitochondrial Activity

The JC-1 mitochondrial membrane potential assay kit (C2003S; Beyotime Institute of Biotechnology, Shanghai, China) was used to assess mitochondrial activity in spermatozoa. This kit detects mitochondrial membrane potential using a potential-sensitive fluorescent dye that accumulates in mitochondria in a membrane potential-dependent manner. At high membrane potential, the JC-1 dye forms red fluorescent aggregates, whereas at low potential, it exists as green monomers. Flow cytometry (BeamCyte, Changzhou, China) was employed to measure the fluorescence intensities of the red and green channels, and the ratio of red to green fluorescence was used as an indicator of mitochondrial membrane potential and overall mitochondrial activity [34]. Each treatment group was performed with three independent biological replicates (*n* = 3).

### 2.10. Evaluation of Sperm ROS Content

Intracellular ROS levels in spermatozoa were quantified using a ROS measurement kit (M36008, Thermo Fisher Scientific, Shanghai, China) [35]. This kit employs a fluorescent probe that reacts with ROS within the spermatozoa, resulting in fluorescence emission. The fluorescence intensity is proportional to the amount of ROS present, enabling precise quantification of oxidative stress in the spermatozoa. Flow cytometry was used to measure fluorescence intensity. We used the FL1 channel for detection. Each treatment group was performed with three independent biological replicates (*n* = 3).

### 2.11. Measurement of Sperm MDA Levels

The MDA assay kit (EATP100, Nanjing Jiancheng Institute of Biotechnology, China) was used to measure sperm MDA levels according to the manufacturer’s instructions and previous studies [36]. The samples were subjected to ultrasonic disruption in an ice bath, followed by thorough mixing with the prepared reaction buffer. The mixture was then incubated in a boiling water bath for 40 min. After extraction, it is cooled by running water, and the samples were centrifuged to obtain the supernatant, which was subsequently analyzed by measuring absorbance at 532 nm using a microplate reader (ZEISS DM200LED, Oberkochen, Germany). Each treatment group was performed with three independent biological replicates (*n* = 3).

### 2.12. Measurement of Sperm LPO Levels

The lipid peroxidation (LPO) assay kit (A160-1, Nanjing Jiancheng Institute of Biotechnology, Nanjing, China) was used to measure sperm LPO levels [37]. The samples were subjected to ultrasonic disruption in an ice water bath (power: 300 W, with 3 to 5 s pulses and repeated 3 to 5 times). After mixing with the appropriate reagents, each sample was incubated at 45 °C for 60 min. Following incubation, the samples were centrifuged to collect the supernatant, and absorbance at 586 nm was measured using a microplate reader. Each treatment group was performed with three independent biological replicates (*n* = 3).

### 2.13. Measurement of MBOAT2 Enzymatic Activity

Measurement of MBOAT2 enzyme activity using an MBOAT2 assay kit (JM-08154S2, Jiangsu Jingmei Institute of Bioengineering, Suzhou, China). The prepared samples and standards were incubated at 37 °C for 30 min. After washing, an enzyme-labeled reagent was added, followed by a second incubation at 37 °C for 30 min. Subsequently, a chromogenic substrate solution was added for color development. After 10 min, a stop solution was added. Absorbance values were quantified using a microplate reader (ZEISS DM200LED, Oberkochen, Germany). Each treatment group was performed with three independent biological replicates (*n* = 3).

### 2.14. Measurement of Ferrous Iron and Total Iron Contents

The content of Fe^2+^ was measured using a ferrous iron colorimetric assay kit (E-BC-K881-M, Elabscience, Wuhan, China) as per the manufacturer’s protocol. The content of Fe^2+^ and Fe^3+^ was measured using a total iron colorimetric assay kit (E-BC-K772-M, Elabscience, Wuhan, China) [38]. Following ice-cold homogenization and lysis for 10 min, the supernatant was collected by centrifugation (12,000 rpm, 4 °C, 15 min), combined with designated reagents according to the manufacturer’s instructions, and incubated at 37 °C for 30 min. Absorbance values were quantified at 593 nm using a microplate reader. Each treatment group was performed with three independent biological replicates (*n* = 3).

### 2.15. Immunofluorescence Localization

Post-fixation and permeabilization, samples were blocked with 10% goat serum-containing buffer, followed by sequential incubation with primary antibodies (overnight at 4 °C) and fluorophore-conjugated secondary antibodies (light-protected). Distinct spatial distribution patterns were observed: MBOAT2 signals localized to both acrosomal and flagellar regions, while ACSL3 exhibited exclusive acrosomal enrichment of primary antibodies. Such as anti-MBOAT2 (bs-18709R, Bioss, Beijing, China) and anti-ACSL3 (DF9606, AffinitY, Liyang, China). Each treatment group was performed with three independent biological replicates (*n* = 3).

### 2.16. Western Blotting

Following lysis buffer incubation, sperm suspensions were subjected to differential centrifugation at 12,000 rpm for 15 min (4 °C). This clarification effectively separated cellular debris from soluble protein fractions, and clarified supernatants were aliquoted and stored at −80 °C prior to electrophoretic analysis. Equivalent protein aliquots were resolved on 10% SDS-PAGE gels under reducing conditions and transferred to PVDF membranes at 120 V for 90 min. Membranes were blocked with 5% TBST (Tris-buffered saline containing 0.1% Tween-20) for 2 h at 25 °C, followed by overnight incubation at 4 °C with primary antibodies. After TBST washes, membranes were incubated with HRP-conjugated secondary antibodies for 1 h at 25 °C. Images were captured using a fluorescence microscope (ZEISS DM200LED, Oberkochen, Germany) and quantified using ImageLab™ software (ImageJ 2 Fiji 1.54p). Each treatment group was performed with three independent biological replicates (*n* = 3). Primary antibodies such as anti-ACSL3 (DF9606, AffinitY, Jiangsu, China), anti-ACSL4 (ABclonal, Wuhan, China), anti-GPX4 (ET1706-45, HUABIO, Hangzhou, China), and anti-SLC7A11 (A2413, ABclonal, Wuhan, China).

### 2.17. Statistical Analysis

Data from three independent replicates were compared using one-way ANOVA followed by Tukey’s post hoc test (Statview; Abacus Concepts, Inc., Berkeley, CA, USA). Prior to parametric testing, the distribution of each parameter was evaluated for normality using the Shapiro–Wilk test in SPSS (IBM SPSS Statistics 27), and the homogeneity of variances was verified. When normality and homogeneity are satisfied (*p* > 0.05), data are presented as mean ± SEM.

## 3. Results

### 3.1. Addition of OA Improved Sperm Motility Parameters

As illustrated in Table 1, computer-assisted sperm analysis (CASA) revealed significant improvements in sperm kinematic parameters during refrigerated storage at 4 °C. The addition of 1 mM OA demonstrated marked enhancement in both TM and PM at critical storage intervals of 48 h and 72 h (*p* < 0.05). Additionally, OA significantly (*p* < 0.05) enhanced VCL, VSL, VAP, LIN, STR, and WOB parameters at 24 h. In addition, the 1 mM OA group showed significantly higher total motility than the control group after 96 h of storage. Notably, baseline assessments conducted immediately after OA treatment (0 h) showed comparable sperm kinematic parameters across all experimental groups (*p* < 0.05), confirming the temporal specificity of the observed chilled storage effects.

### 3.2. Addition of OA Improved the Sperm Acrosomal Integrity and Viable Spermatozoa Preserved

**Figure 1:**
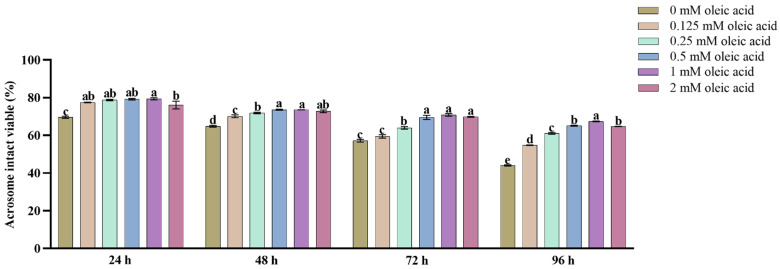
Acrosomal integrity and viable sperm of goat spermatozoa preserved at 4 °C were quantitatively assessed to evaluate the effects of graded OA concentrations. Data are expressed as means ± SEM from triplicate biological replicates (*n* = 3). Different lowercase letters indicate statistically significant differences (*p* < 0.05).

As depicted in Figure 1, control group spermatozoa exhibited significant plasma membrane deterioration during 4 °C preservation. OA-supplemented treatment groups (0.25, 0.5, 1, and 2 mM) demonstrated concentration-dependent membrane stabilization effects. Notably, the 1 mM OA treatment showed superior cryoprotective efficacy at 96 h storage, with statistically distinct superscripts (Figure 1) indicating significant inter-group differences (*p* < 0.05).

### 3.3. Addition of OA Improved Sperm Mitochondrial Activity

**Figure 2:**
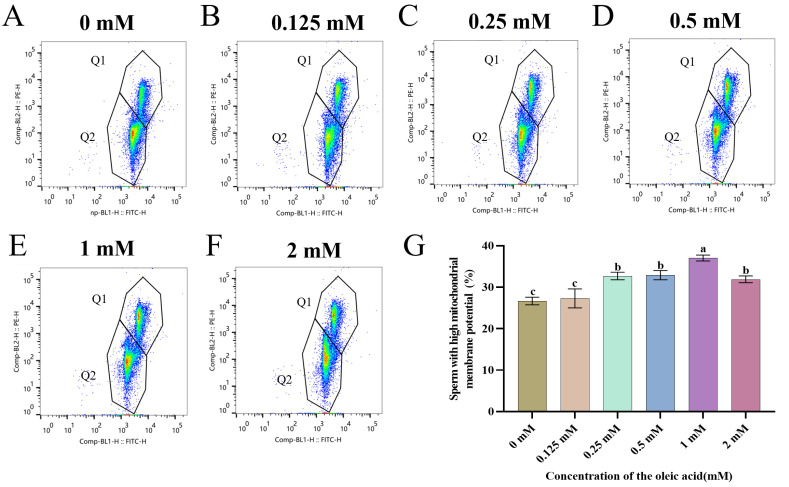
Effect of OA supplementation on mitochondrial membrane potential in chilled stored goat sperm. (**A**–**F**) Assessment of mitochondrial transmembrane potential in goat sperm after 4 °C preservation with graded OA concentrations. The designated Q1 region refers to sperm with high mitochondrial membrane potential, while Q2 region refers to sperm with low mitochondrial membrane potential. As the color deepens from blue → green → yellow → red, the number of sperm cells within that region increases. (**G**) Quantitative comparison of sperm subpopulations with high MMP. Data are expressed as means ± SEM from triplicate biological replicates (*n* = 3). Different lowercase letters indicate statistically significant differences (*p* < 0.05).

All OA-supplemented groups exhibited concentration-dependent enhancement of mitochondrial functionality compared to untreated controls (Figure 2A–F). Notably, the 1 mM OA treatment demonstrated maximal mitochondrial polarization preservation, with values significantly exceeding other concentrations (*p* < 0.05) (Figure 2G).

### 3.4. OA Attenuates Chilling-Induced Oxidative Damage in Goat Sperm by Suppressing ROS Generation and Lipid Peroxidation

**Figure 3:**
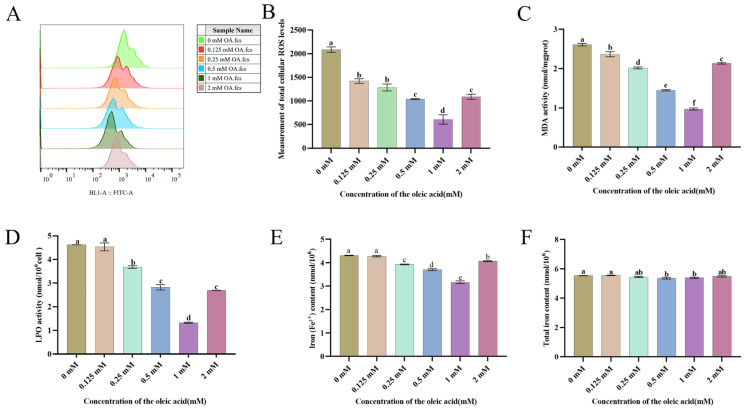
Delineates the effects of oleic acid (OA) on the antioxidant capacity of goat sperm during 4 °C preservation. (**A**,**B**) Impact of oleic acid on reactive oxygen species (ROS) levels in goat sperm during 4 °C cold storage. (**C**) Effect of different concentrations of OA on sperm MDA. (**D**) Effect of different concentrations of OA on sperm LPO. (**E**) Effect of different concentrations of OA on sperm Fe^2+^. (**F**) Effect of different concentrations of OA on sperm total iron. Data are expressed as means ± SEM from triplicate biological replicates (*n* = 3). Different lowercase letters indicate statistically significant differences (*p* < 0.05).

To elucidate the mechanism underlying OA-mediated attenuation of lipid peroxidation via ACSL3 pathway activation, mitochondrial ROS generation was quantitatively analyzed in cryopreserved spermatozoa. The OA-supplemented group demonstrated dose-responsive ROS suppression compared to untreated controls. As demonstrated in the corresponding Figure, 1 mM OA treatment elicited significant ROS suppression compared to untreated controls (*p* < 0.05) (Figure 3A,B).

To investigate the protective effects of OA on goat sperm preserved at 4 °C, we evaluated two key oxidative stress biomarkers: MDA levels and LPO. As illustrated in Figure 3C, OA-treated groups demonstrated significant mitigation of oxidative damage compared to controls, with particularly notable decreases in MDA content. Quantitative analysis revealed that 1 mM OA treatment induced the most pronounced reduction in MDA levels (Figure 3C). This protective effect was further confirmed through LPO measurements, where 1 mM OA supplementation resulted in a significant decrease in sperm lipid peroxidation (Figure 3D). Furthermore, all experimental groups except the 0.125 mM OA cohort showed marked reductions in LPO and MDA levels, with statistical significance confirmed at *p* < 0.05.

Ferrous iron is a key regulator in the process of ferroptosis. Its accumulation can catalyze ROS generation via the Fenton reaction, exacerbating lipid peroxidation. In this study, analysis of iron metabolism in sperm revealed that the 1 mM oleic acid treatment group not only significantly reduced Fe^2+^ content (*p* < 0.05) but also showed a downward trend in total iron levels (*p* < 0.05) (Figure 3E,F).

### 3.5. Localization and Expression of ACSL3 and MBOAT2 Pathways in Goat

**Figure 4:**
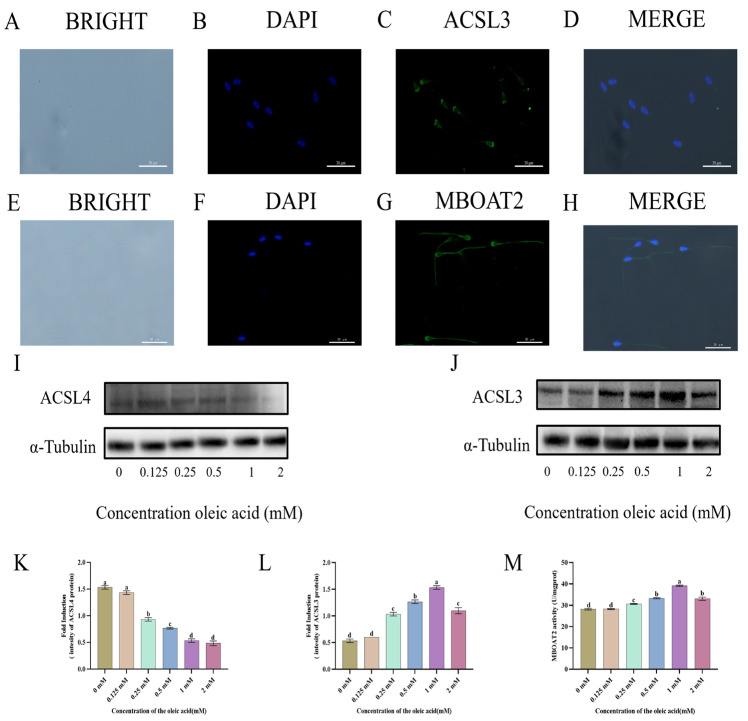
Subcellular localization of ACSL3 (**A**–**D**) and MBOAT2 (**E**–**H**) by immunofluorescence and expression analysis of ACSL4 (**I**) and ACSL3 (**J**) in goat sperm. (**K**,**L**) Quantitative analysis of ACSL4 and ACSL3. (**M**) Enzymatic activity of MBOAT2 in sperm. ACSL3: Acyl-CoA Synthetase Long Chain Family Member 3. ACSL4: Acyl-CoA Synthetase Long Chain Family Member 4. MBOAT2: Membrane Bound Glycerophospholipid O-Acyltransferase 2. Data are expressed as means ± SEM from triplicate biological replicates (*n* = 3). Different lowercase letters indicate statistically significant differences (*p* < 0.05).

MBOAT2, a membrane-bound O-acyltransferase, is preferentially localized and concentrated in the acrosomal and flagellar regions of goat sperm (Figure 4E–H). In contrast, ACSL3, a long-chain fatty acid coenzyme A ligase critical for fatty acid activation, is specifically localized to the acrosome of caprine sperm, where it plays a crucial role in lipid metabolic processes (Figure 4A–D).

To elucidate the mechanism by which oleic acid (OA) enhances sperm quality, we assessed the protein expression levels of ACSL3 and ACSL4 in spermatozoa (Figure 4I,J, Supplementary Figures S1 and S2). ACSL3 expression was significantly regulated following treatment with 0.25, 0.5, 1, and 2 mM OA (*p* < 0.05), whereas the increase observed with 0.125 mM OA was not statistically different from the control (Figure 4L). Moreover, exposure to 1 mM OA resulted in a marked reduction in the total protein level of ACSL4 (Figure 4K). More so, MBOAT2 enzyme activity was significantly higher in the 1 mM oleic acid group relative to the control (Figure 4M).

### 3.6. The Expression of GPX4 and SLC7A11 Proteins

**Figure 5:**
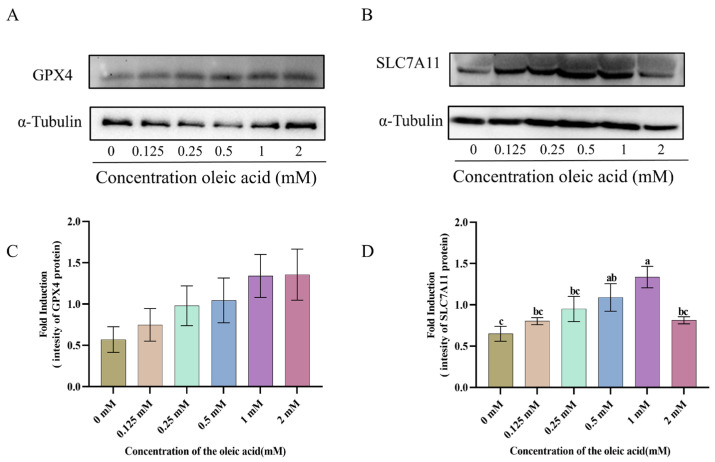
Western blot analysis of GPX4 and SLC7A11 expression in goat sperm. (**A**,**B**) Detection of GPX4 and SLC7A11 protein expression in goat sperm. (**C**,**D**) Quantitative analysis of GPX4 and SLC7A11. Data normalized to α-Tubulin loading control, presented as mean ± SEM (*n* = 3). Different lowercase letters indicate statistically significant differences (*p* < 0.05).

To investigate the effect of oleic acid (OA) on the key ferroptosis-related pathway SLC7A11/GPX4, we examined the protein expression levels of GPX4 and SLC7A11 in spermatozoa (Figure 5A,B, Supplementary Figures S3 and S4). Compared with the control group, treatment with 0.125, 0.25, 0.5, 1 and 2 mM OA significantly increased the expression levels of both GPX4 and SLC7A11 (*p* < 0.05). Notably, treatment with 1 mM OA led to a pronounced elevation in the total protein levels of SLC7A11 and GPX4 (Figure 5C,D).

### 3.7. Oleic Acid Prevents RSL3-Mediated Ferroptosis Through Lipid Peroxidation Inhibition in Sperm Cells

**Figure 6:**
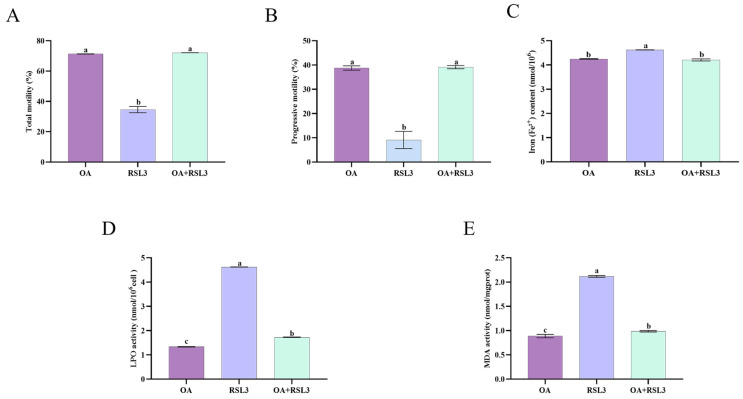
Co-incubation with oleic acid alleviated RSL3-induced ferroptosis impairment of sperm progressive motility (**A**,**B**). Supplementation with oleic acid (OA) in the presence of RSL3 significantly reduced cellular ferrous iron uptake (**C**), thereby decreasing LPO (**D**) and MDA (**E**) levels. Data are presented as mean ± SEM (*n* = 3). Different lowercase letters indicate statistically significant differences (*p* < 0.05).

The RSL3 group exhibited a significant reduction in motility, whereas OA co-treatment markedly restored motility levels. Co-incubation with oleic acid markedly attenuated the RSL3-induced decline in sperm motility associated with ferroptosis (*p* < 0.05) (Figure 6A,B). Compared to the RSL3 group, OA co-incubation markedly attenuated the accumulation of Fe^2+^, LPO, and MDA.

## 4. Discussion

Recent advancements in chilled storage technology have significantly improved the viability of goat sperm. However, cold-induced ferroptosis, mediated by iron overload, oxidative stress, and lipid peroxidation, remains a critical factor compromising sperm survival [25]. Optimizing cryoprotectant formulations has thus emerged as a pivotal strategy to mitigate this damage [39]. Our findings demonstrate that OA, a monounsaturated fatty acid, specifically activates the ACSL3 signaling pathway, suppresses lipid peroxidation cascades, and inhibits downstream ferroptosis signaling. These actions collectively enhance the kinematic parameters and membrane integrity of sperm in chilled storage conditions.

This study demonstrates that OA confers significant protection against low-temperature-induced ferroptosis in goat sperm. Our findings are consistent with previous work by Cao et al. in tumor models [27], who demonstrated that OA exerts hypothermic effects through dual mechanisms involving oxidative stress mitigation and membrane lipid stabilization, thereby extending sperm viability from 24 h to 96 h under hypothermic conditions (4 °C). The suggestion is that its cytoprotective role may be conserved across cell types. The observed dose-dependent improvement in sperm motility (0.125–1 mM OA) suggests that OA facilitates optimal energy metabolism and stabilizes membrane dynamics, thereby helping sperm resist hypothermic stress. CASA analysis (including parameters such as VCL, VSL, and LIN) confirmed that OA supplementation significantly improved sperm motility, implying that OA maintains the structural and functional integrity of sperm by mitigating lipid peroxidation and ferroptosis damage. While 2 mM OA treatment exhibited some beneficial effects on total and progressive motility, the impaired kinematic parameters observed suggest potential dose-dependent cytotoxicity, possibly due to OA-induced metabolic overload. However, this phenomenon was not further investigated in the current study.

Additionally, OA treatment improved sperm membrane integrity, as confirmed by PI and FITC-PNA staining. The integrity of both the acrosomal and plasma membranes is closely associated with oxidative stress dynamics and the lipid composition of the membrane. While polyunsaturated fatty acids (PUFAs) enhance membrane fluidity, excessive incorporation-particularly within phosphatidyl ethanolamine species-renders membranes highly susceptible to lipid peroxidation cascades. It is worth noting that a concentration of 2 mM OA led to a reduction in mitochondrial membrane potential, indicating a potential cytotoxic effect at higher doses. Wang et al. [40] observed that elevated OA concentrations led to a marked decline in oxygen consumption and ATP generation, prompting cells to rely more heavily on glycolysis for energy production. This mitochondrial impairment intensifies ROS accumulation and energetic imbalance, ultimately heightening the susceptibility to ferroptosis and related oxidative cell death pathways. This further indicates the damage caused by high concentrations of oleic acid to mitochondria. Leakage from the mitochondrial electron transport chain generates reactive oxygen species (ROS), which further propagate lipid peroxidation, with MDA serving as both a terminal byproduct and a biomarker of oxidative membrane damage. OA, as a monounsaturated fatty acid, likely preserves membrane integrity under hypothermic stress by stabilizing lipid organization and decreasing the susceptibility of PUFA-rich domains to oxidation.

Notably, MUFAs maintain lipid homeostasis through enzyme-mediated remodeling processes. Specifically, MBOAT2 collaboratively mediates PE remodeling, strategically reducing PUFAs occupancy at the sn-2 position to minimize susceptibility to peroxidation. In goat sperm chilled storage, the inherent enrichment of membrane PUFAs creates heightened oxidative vulnerability under hypothermic stress. Previous research in mice has demonstrated that oleic acid modulates cellular lipid composition and protects from iron overload (FAC)-induced hepatic lipid peroxidation and injury [26]. Our data demonstrates that OA supplementation significantly attenuated ROS production (*p* < 0.05), lipid peroxidation, and MDA accumulation compared to untreated controls during 4 °C preservation (*n* = 3), indicating potent oxidative stress mitigation. These findings are consistent with previous research, adding further evidence that MUFA supplementation preferentially suppresses ROS generation through lipid raft stabilization rather than via direct radical scavenging.

This study demonstrates that, in addition to enhancing sperm viability, OA reduces the expression of ferroptosis-related markers such as ACSL4, GPX4, and SLC7A11, underscoring its effectiveness in mitigating oxidative damage. Notably, at a concentration of 1 mM, OA markedly reduced iron accumulation and inhibited lipid peroxidation—two key biochemical hallmarks of ferroptosis—through the regulation of redox homeostasis and iron metabolism pathways. Interestingly, in certain cancer cell models, oleic acid has also been shown to promote ferroptosis rather than suppress it. For instance, a study in A549 and H1299 lung cancer cells reported that OA treatment led to the upregulation of ACSL4 and downregulation of GPX4, accompanied by elevated ROS levels and enhanced lipid peroxidation, ultimately accelerating ferroptosis cell death [41]. These findings suggest that the role of OA in ferroptosis is highly context dependent. Under normal physiological conditions, OA tends to exert a cytoprotective effect by stabilizing membranes and reducing oxidative burden; however, in malignant or metabolically reprogrammed cells, OA may act as a pro-ferroptosis “lethal agent,” exploiting altered lipid metabolism to trigger cell death [41].

To further validate the protective mechanism of OA, we employed an RSL3-induced ferroptosis model. Results demonstrated that sperm motility in the RSL3 plus OA co-treatment group was significantly restored, and levels of lipid peroxidation markers (MDA and LPO) were markedly reduced, indicating that OA effectively alleviated RSL3-induced ferroptosis damage. Mechanistically, RSL3 induces ferroptosis by inhibiting GPX4, resulting in fatty acid peroxidation and mitochondrial damage [42]. The mitigation observed upon OA supplementation implies a competitive remodeling of the membrane lipid pool. As a monounsaturated fatty acid, OA likely competes for incorporation into membrane phospholipids, particularly PE, thereby reducing the synthesis of PUFA-PE and subsequent lipid peroxidation. This mechanism aligns with the “MUFA-PE replacement hypothesis” [43], which posits that MUFA enrichment confers structural resistance to oxidative degradation. Collectively, these findings support the notion that OA preserves sperm function under oxidative challenge primarily through targeted lipid remodeling rather than nonspecific antioxidant action.

## 5. Conclusions

In conclusion, this study demonstrates that OA effectively protects goat sperm from ferroptosis-induced damage under low-temperature conditions. OA modulates ferroptosis-associated pathways and reduces oxidative stress, indicating its potential as a therapeutic candidate for cryopreservation protocols. Its dose-dependent effect underscores the importance of optimizing OA concentration to achieve maximal protective efficacy. These findings contribute to a deeper understanding of the molecular mechanisms underlying sperm preservation and offer valuable insights into advancing reproductive technologies in caprine species. Future studies should investigate the long-term effects of OA supplementation on fertility outcomes and evaluate its applicability across other livestock species under diverse environmental stressors.

## Figures and Tables

**Table 1:** Effect of OA on goat sperm motility parameters measured with CASA during storage.

Sperm Parameters	Time (h)	0 mM	0.125 mM	0.25 mM	0.5 mM	1 mM	2 mM
Total motility (%)	24	80.2 ± 1.1	80.9 ± 1.1	80.3 ± 1.1	80.7 ± 0.1	81.4 ± 0.7	79.8 ± 0.7
	48	71.1 ± 0.2 ^c^	72.8 ± 0.6 ^bc^	73.5 ± 0.7 ^b^	73.5.9 ± 0.3 ^b^	78.5 ± 1.0 ^a^	74.3 ± 0.7 ^b^
	72	70.8 ± 0.3 ^e^	70.8 ± 0.3 ^e^	73.4 ± 0.5 ^c^	75.2 ± 0.2 ^b^	76.5 ± 0.5 ^a^	71.9 ± 0.1 ^d^
	96	60.5 ± 0.9^d^	64.5 ± 1.4 ^cd^	66.0 ± 0.3 ^bc^	70.4 ± 3.3 ^ab^	72.5 ± 0.2 ^a^	66.6 ± 1.1 ^bc^
Progressive motility (%)	24	47.9 ± 1.5 ^bc^	48.2 ± 1.1 ^bc^	49.8 ± 1.9 ^bc^	51.2 ± 1.8 ^ab^	55.6 ± 1.8 ^a^	45.8 ± 1.3 ^c^
	48	43.8 ± 2.6	47 ± 0.2	47.4 ± 1.7	50.2 ± 3.4	50.5 ± 4.6	40.5 ± 1.8
	72	40.8 ± 0.5 ^b^	42.1 ± 1.7 ^b^	44.7 ± 1.3 ^ab^	45.5 ± 0.6 ^ab^	50.2 ± 4.9 ^a^	39.8 ± 0.9 ^b^
	96	30.5 ± 2.5	34.6 ± 3.7	35.5 ± 2.0	37.2 ± 0.9	37.7 ± 3.6	31.8 ± 2.3
VCL (μm/s)	24	109.5 ± 14.5 ^b^	111.8 ± 13.5 ^b^	125.3 ± 10.5 ^ab^	139.9 ± 5.4 ^ab^	156.3 ± 1.2 ^a^	147.6 ± 10.4 ^a^
	48	108.8 ± 8.7 ^d^	112.4 ± 1.2 ^cd^	124.5 ± 3.9 ^bc^	131.5 ± 2.9 ^ab^	140.0 ± 2.3 ^a^	127.2 ± 0.4 ^ab^
	72	110.8 ± 10.2	115.4 ± 11.1	124.9 ± 6.7	134.4 ± 4.6	138.9 ± 3.9	112.8 ± 9.4
	96	111.2 ± 9.0	132.5 ± 9.3	124.5 ± 11.8	113.9 ± 10.3	134.6 ± 4.1	117.6 ± 6.1
VSL (μm/s)	24	49.4 ± 9.0 ^c^	48.7 ± 7.8 ^c^	60.4 ± 4.7 ^ab^	65.6 ± 2.8 ^ab^	75.0 ± 1.3 ^a^	63.0 ± 5.3 ^ab^
	48	48.7 ± 5.5 ^b^	49.6 ± 0.2 ^b^	63.3 ± 2.5 ^a^	63.9 ± 4.4 ^a^	64.0 ± 4.9 ^a^	57.6 ± 0.6 ^ab^
	72	50.9 ± 4.4 ^b^	53.8 ± 7.4 ^b^	60.9 ± 3.1 ^ab^	63.6 ± 0.5 ^ab^	71.2 ± 3.4 ^a^	50.9 ± 4.3 ^b^
	96	50.3 ± 2.7	55.2 ± 3.3	57.9 ± 4.8	48.5 ± 3.6	59.0 ± 2.6	49.0 ± 3.5
VAP (μm/s)	24	63.4 ± 9.1 ^b^	63.1 ± 7.9 ^b^	73.7 ± 5.7 ^ab^	79.7 ± 3.3 ^ab^	90.9 ± 0.5 ^a^	80.0 ± 6.0 ^ab^
	48	65.2 ± 4.9 ^c^	68.6 ± 3.5 ^bc^	73.0 ± 2.0 ^abc^	76.6 ± 4.5 ^ab^	80.1 ± 0.5 ^a^	71.6 ± 0.3 ^abc^
	72	64.4 ± 5.2 ^b^	66.5 ± 7.1 ^b^	73.2 ± 3.5 ^ab^	77.5 ± 0.7 ^ab^	84.9 ± 1.7 ^a^	63.9 ± 4.6 ^b^
	96	61.4 ± 4.2	69.5 ± 3.9	70.5 ± 6.1	61.3 ± 4.6	74.2 ± 2.0	61.7 ± 3.6
BCF (Hz)	24	32.8 ± 2.0 ^a^	32.6 ± 1.0 ^a^	26.5 ± 0.3 ^b^	30.0 ± 0.9 ^ab^	29.3 ± 0.4 ^ab^	28.0 ± 1.9 ^b^
	48	32.1 ± 2.5 ^a^	28.8 ± 1.2 ^bc^	29.2 ± 0.5 ^bc^	26.6 ± 1.7 ^b^	29.5 ± 0.1 ^bc^	29.2 ± 0.2 ^bc^
	72	33.6 ± 2.4 ^a^	30.4 ± 0.6 ^ab^	28.4 ± 1.2 ^ab^	30.3 ± 0.4 ^ab^	27.2 ± 1.1 ^b^	30.5 ± 2.4 ^ab^
	96	22.4 ± 1.1 ^b^	23.2 ± 1.4 ^b^	28.9 ± 1.4 ^a^	23.8 ± 1.2 ^b^	29.1 ± 0.8 ^a^	23.1 ± 0.1 ^b^
LIN (%)	24	43.3 ± 2.9 ^bc^	42.1 ± 2.1 ^c^	48.9 ± 0.5 ^a^	47.5 ± 0.9 ^ab^	49.2 ± 0.8 ^a^	43.4 ± 0.7 ^bc^
	48	43.3 ± 1.2	44.9 ± 0.2	46.6 ± 1.2	47.3 ± 2.7	47.8 ± 2.5	43.3 ± 0.6
	72	44.0 ± 0.8	45.6 ± 2.8	48.6 ± 1.9	47.9 ± 1.4	51.7 ± 3.8	44.2 ± 1.2
	96	45.5 ± 1.3	42.0 ± 0.9	46.4 ± 0.5	43.2 ± 2.9	43.9 ± 2.5	41.8 ± 0.8
STR (%)	24	73.8 ± 3.6 ^bc^	73.5 ± 3.4 ^c^	81.0 ± 0.2 ^ab^	81.0 ± 0.3 ^ab^	82.3 ± 1.3 ^a^	77.7 ± 0.6 ^abc^
	48	73.2 ± 2.9 ^b^	76.7 ± 0.8 ^ab^	81.5 ± 1.1 ^a^	82.0 ± 1.3 ^a^	80.5 ± 1.9 ^a^	78.9 ± 0.6 ^a^
	72	74.7 ± 1.5	77.8 ± 3.3	81.0 ± 1.6	80.8 ± 1.0	82.4 ± 2.5	76.4 ± 2.5
	96	81.2 ± 1.5	78.6 ± 0.9	80.4 ± 0.5	78.3 ± 3.3	77.6 ± 2.6	78.2 ± 0.9
WOB (%)	24	57.2 ± 1.1 ^ab^	55.9 ± 0.5 ^bc^	59.3 ± 0.5 ^a^	57.5 ± 1.0 ^ab^	58.9 ± 0.3 ^a^	54.8 ± 0.4 ^c^
	48	56.6 ± 0.08	56.7 ± 0.4	56.2 ± 0.2	56.6 ± 0.7	58.2 ± 1.8	56.2 ± 0.4
	72	57.2 ± 0.6	57.2 ± 1.3	58.8 ± 1.4	58.2 ± 1.4	61.6 ± 2.9	56.4 ± 0.2
	96	55.3 ± 0.7 ^ab^	53.1 ± 0.8 ^b^	56.8 ± 0.5 ^a^	54.3 ± 1.5 ^ab^	55.4 ± 1.5 ^ab^	52.8 ± 0.4 ^b^

Values are expressed as mean ± standard error. Different letters within the same row indicate significant differences (*p* < 0.05), *n* = 3. VCL, curvilinear velocity; VSL, straight-line velocity; VAP, average path velocity; BCF, beat-cross frequency; LIN, linearity (VSL/VCL); STR, straightness (VSL/VAP); WOB, wobble (VAP/VCL).

## Data Availability

Upon reasonable request, the datasets of this study can be made available from the corresponding author.

## Supplementary Materials

The following supporting information can be downloaded at https://www.mdpi.com/article/10.3390/ani15223258/s1: Figure S1: Effect of different concentrations of OA on the ACSL4 protein in goat sperm; Figure S2: Effect of different concentrations of OA on the ACSL3 protein in goat sperm; Figure S3: Effect of different concentrations of OA on the GPX4 protein in goat sperm; Figure S4: Effect of different concentrations of OA on the SLC7A11 protein in goat sperm.
