# Supplementary material for: Oleic Acid Improves Goat Sperm Quality by Enhancing the MBOAT2/ACSL3 Pathway to Attenuate Ferroptosis
Source: Animals (Basel). 2025 Nov 10;15(22):3258. doi: 10.3390/ani15223258 (PMC12649600; doi:10.3390/ani15223258)
Supplement: Supplementary file 1 [file animals-15-03258-s001.zip › animals-3946416-supplementary.pdf]

# *Supplementary Figures and legends*

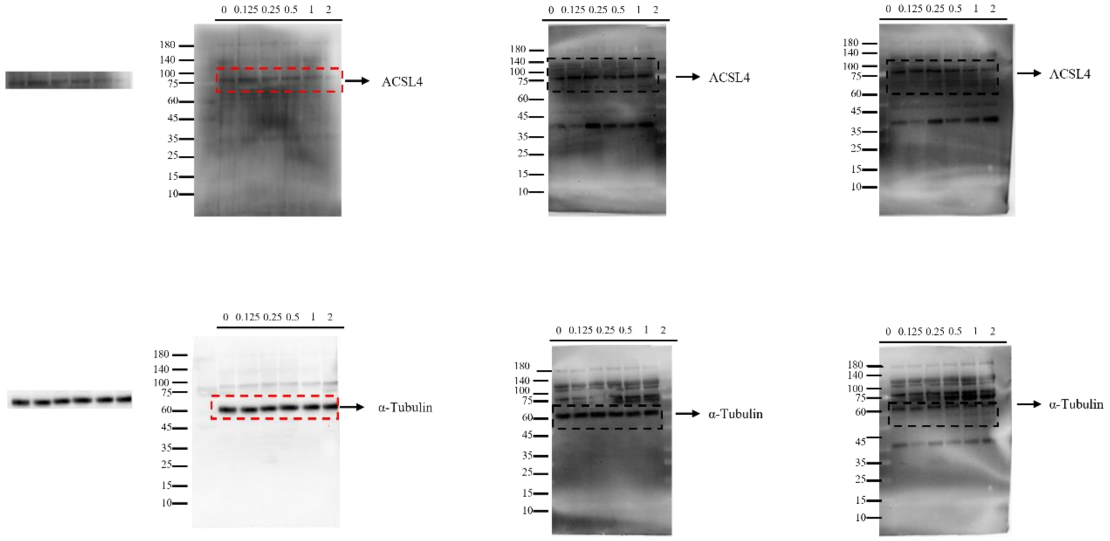


**Supplementary Figure S1.** Effect of different concentrations of OA on the ACSL4 protein in goat sperm.

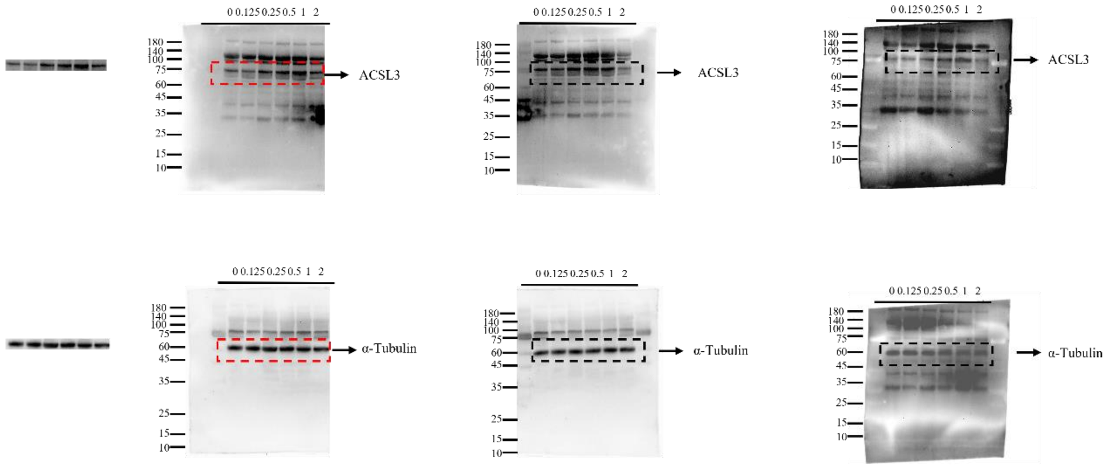


**Supplementary Figure S2.** Effect of different concentrations of OA on the ACSL3 protein in goat sperm.

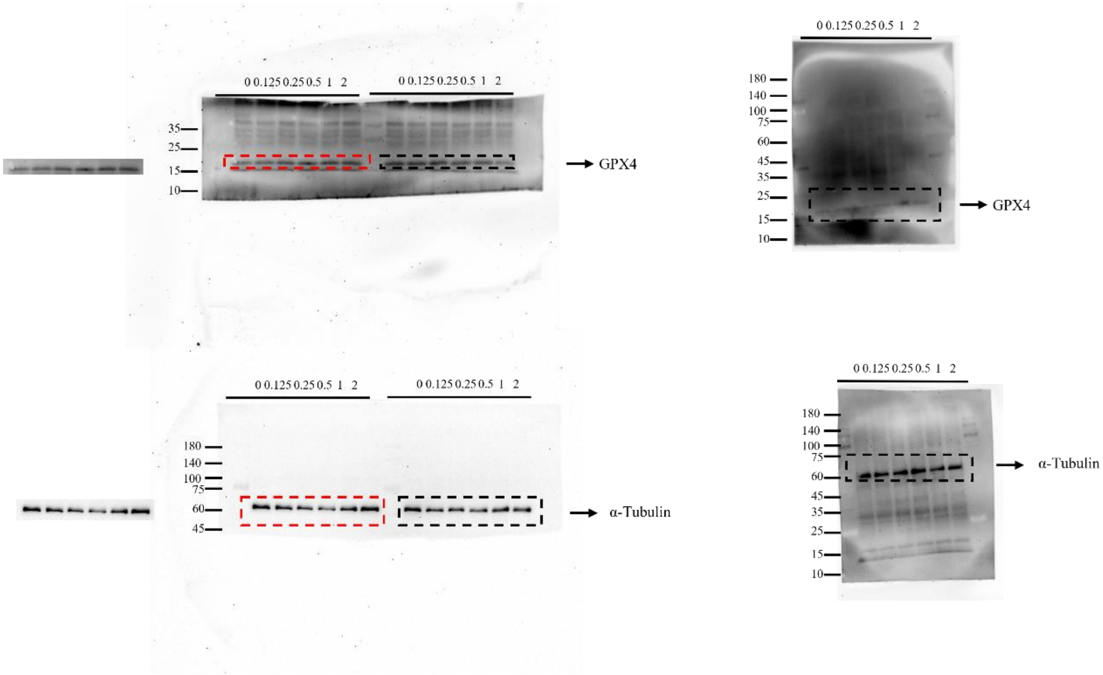


**Supplementary Figure S3.** Effect of different concentrations of OA on the GPX4 protein in goat sperm.

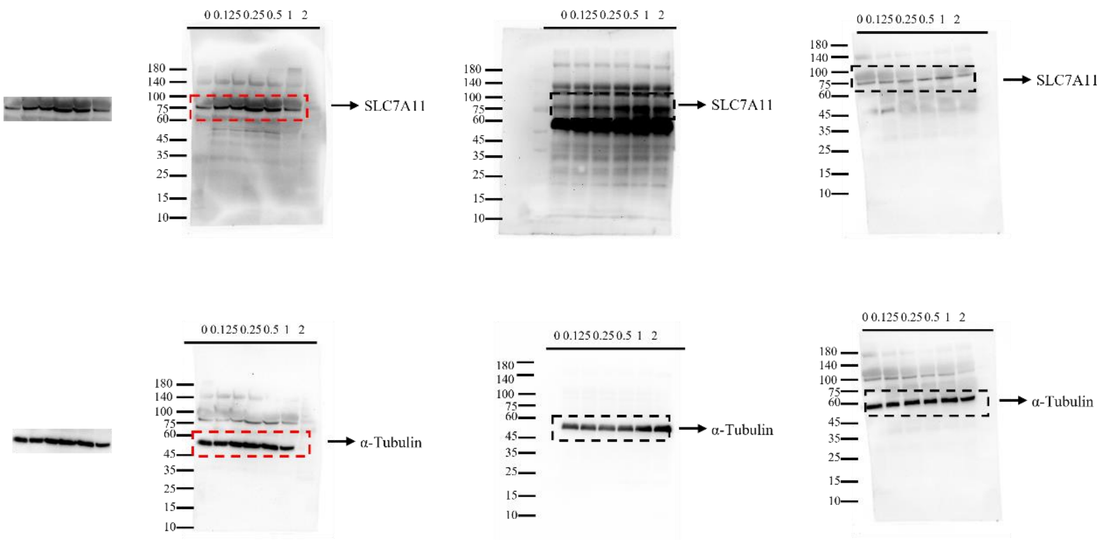


**Supplementary Figure S4.** Effect of different concentrations of OA on the SLC7A11 protein in goat sperm.
